# cudaMap: a GPU accelerated program for gene expression connectivity mapping

**DOI:** 10.1186/1471-2105-14-305

**Published:** 2013-10-11

**Authors:** Darragh G McArt, Peter Bankhead, Philip D Dunne, Manuel Salto-Tellez, Peter Hamilton, Shu-Dong Zhang

**Affiliations:** 1 Centre for Cancer Research and Cell Biology (CCRCB), Queen’s University Belfast (QUB), Belfast, Northern Ireland, UK

## Abstract

**Background:**

Modern cancer research often involves large datasets and the use of sophisticated statistical techniques. Together these add a heavy computational load to the analysis, which is often coupled with issues surrounding data accessibility. Connectivity mapping is an advanced bioinformatic and computational technique dedicated to therapeutics discovery and drug re-purposing around differential gene expression analysis. On a normal desktop PC, it is common for the connectivity mapping task with a single gene signature to take > 2h to complete using sscMap, a popular Java application that runs on standard CPUs (Central Processing Units). Here, we describe new software, cudaMap, which has been implemented using CUDA C/C++ to harness the computational power of NVIDIA GPUs (Graphics Processing Units) to greatly reduce processing times for connectivity mapping.

**Results:**

cudaMap can identify candidate therapeutics from the same signature in just over thirty seconds when using an NVIDIA Tesla C2050 GPU. Results from the analysis of multiple gene signatures, which would previously have taken several days, can now be obtained in as little as 10 minutes, greatly facilitating candidate therapeutics discovery with high throughput. We are able to demonstrate dramatic speed differentials between GPU assisted performance and CPU executions as the computational load increases for high accuracy evaluation of statistical significance.

**Conclusion:**

Emerging ‘omics’ technologies are constantly increasing the volume of data and information to be processed in all areas of biomedical research. Embracing the multicore functionality of GPUs represents a major avenue of local accelerated computing. cudaMap will make a strong contribution in the discovery of candidate therapeutics by enabling speedy execution of heavy duty connectivity mapping tasks, which are increasingly required in modern cancer research. cudaMap is open source and can be freely downloaded from http://purl.oclc.org/NET/cudaMap.

## Background

Cancer research is becoming extremely high throughput, using modern methods from microarray to next generation sequencing, where thousands of counts of biological entities are collected in order to address complicated biological questions. Gene expression connectivity mapping is a powerful bioinformatic technique for establishing the connections among genes, drugs and diseases [1]. There are three key components in the connectivity mapping process: a gene signature representing the biological state of a researcher’s interest, a core database of reference gene expression profiles for a large number small molecule compounds, and a similarity metric quantifying the closeness of the connection between the gene signature and the reference profiles. Using a common “vocabulary” of gene expression profiling [2], connectivity mapping allows the establishment/discovery of connections between different biological states via their gene expression patterns or characteristics, with great potential for application in areas like predictive toxicity [3], candidate therapeutics discovery and drugs repositioning [4,5]. The systematic microarray experiments by the Broad Institute using different compounds, dosages, and cell lines led to the accumulation of drug expression profiles and laid down the foundation for subsequent data exploration and the development of new frameworks. Following the introduction of an improved connectivity mapping framework featuring more principled statistical testing procedures and increased sensitivity [6], a standalone Java application, sscMap, was developed and released implementing this new algorithm [7], and later enabled with gene signature perturbation capability [8]. Given a user-supplied query gene signature, sscMap calculates a connection score between the query signature and each of the reference gene-expression profiles in the core database. The program also computes similar scores for a large number of randomly generated gene signatures, and compares the observed score with the distribution of random scores to obtain a p-value. The generation and processing of numerous random signatures is the most time consuming part of the computation, and causes a typical run of sscMap on a standard desktop computer to take around ∼2h to complete. Using a larger number of random signatures can lead to more accurate estimations of p-values, but at a cost of requiring overnight or over-weekend runs. Since the computational costs will only increase as the number of reference profiles in the database grows, the speed of connectivity mapping will therefore soon become a limiting factor to its wider application. On the other hand, the tasks involved in connectivity mapping are particularly amenable to parallel processing. This has the potential to lead to a significant reduction in processing times required, thereby not only expanding its capacity for wider and novel applications in cancer research, but also allowing a more accurate estimation of statistical significance when desirable. In recent years, Graphics Processing Unit (GPU) computing has enabled significant advances in several bioinformatic research areas, eg, the MUMmerGPU [9] which allows increased processing speeds in sequence alignment, and CFMDS [10], a software platform for fast dimensionality reduction of genome-scale data. Utilising NVIDIA’s parallel computing architecture CUDA (Compute Unified Device Architecture) we sought to advance the connectivity mapping capabilities by using multiprocessor technology to harness localised, low cost parallel computing.

## Implementation

We installed the CUDA development toolkit on a selection of NVIDIA-enabled computers along with the original sscMap software. These machines had various operating systems and differing processing power coupled with varying CUDA-enabled GPU cards. These cards have different numbers of available programmable cores, ranging from 8 to 448, allowing different numbers of threads that may be run simultaneously (Table 1). This allows us to demonstrate the speed and flexibility afforded by this parallel architecture.

**Table 1:** Machines used in analysis

**Machines and operating systems used in analysis**	**GPUs associated with that machine**
Optiplex 760 Core2 Duo E8500 and 4G of RAM (Windows XP)	no GPU
Armari Magnetar with an Intel I7 processor and 24G of RAM (OpenSuse 12.1)	Tesla C2050, Quadro FX380
Dell Lattitude E5400 with CPU P8700 with 4G of RAM (Windows XP)	Geforce 9200M GS
Armari SM7046GT-TRF with an E5503 processor, 6G of RAM (Windows 7)	Tesla C2050

Three of the four machines had CUDA capable devices and were tested with cudaMap against the sscMap software that ran on the CPU of those machines.

### Design and algorithms

The key quantity in the connectivity mapping framework is the connection score defined between a query gene signature and a refset, i.e., a set of reference gene expression profiles. To evaluate the statistical significance of an observed connection score, a p-value is calculated by comparing the observed score against the distribution of a large number of random scores. These random scores are obtained by generating a large number of random gene signatures following the procedure described in [6], and then calculating the connection score between the same refset and each of the random gene signatures. Additional file 1 provides a more detailed description of the algorithm used in sscMap in the form of pseudocode. Because the original sscMap handles all these tasks sequentially, the large number of random gene signatures needed for each p-value estimation lead to its long execution time. cudaMap here tries to tackle this by parallelizing the process and distributing this heavy computational load across the multiple cores of the GPU device.

In the cudaMap application, 6100 reference profiles are stored in a ‘ref-files’ folder, where each file contains a drug-treated cell line with 22283 genes and their associated signed ranks. Query signature files, each containing a list of gene identifiers (Affymetrix HG-U133A probeset IDs) and their corresponding flags to indicate up- or down-regulation, were placed in a ‘queries’ folder. In order to keep the memory footprint small cudaMap processes queries sequentially, by reading them one at a time and running through the reference profiles to measure the scoring metrics for each reference against the query signature. The GPU device is used to generate random signatures of the same length as the original query signature (using NVIDIA’s cuRAND random number generator).

Firstly we can generate a random gene signature of length *m* using *m* random real numbers uniformly distributed between 0 and 1 as *U*[ 0,1), and the algorithm to achieve this is shown on page 3 of the Additional file 1. To generate a large number, say *N*_*rdsigs*_=10000, of random gene signatures of length *m*, we would (only) need *mN*_*rdsigs*_ random numbers with *U*[ 0,1) distribution. Memory space on the device for an array of *mN*_*rdsig*_ real numbers is allocated before a call to the cuRAND library to fill this array with random numbers. The calculation of *N*_*rdsigs*_ random scores is carried out on the CUDA device. The CUDA kernel calls handle the vectorization on the device primarily by the function “__global__ void computeRandomConnectionScores(......)” to parallelize operations across an allowable number of threads, to remove the bottlenecks above. The key algorithms used in cudaMap are described in Additional file 1.

Upon execution of the software through the command terminal, users will be guided by selection choices in respect to the analysis they wish to perform. The flexibility in user ref-files selection choices has been maintained in the cudaMap version in order to allow users to create metrics against individual drugs or distinct drug-cell-dose combinations. Here, users will be prompted to enter their choice of reference-set characterisation, an expected number of false positives to tolerate, and the number of random signatures to generate for each p-value estimation. Default parameters are set if the users wish to proceed without modification, as is the case in the original sscMap.

### Analysis

We tested the software using gene signatures of increasing size, ranging from a small random signature (n =10), to intermediate and larger signatures that have been used in describing sscMap previously, namely HDACs (n =25) and Random02 (n =189). The latter two signatures are available in the original sscMap software and were used as benchmarks for performance and significant compound retrievals. We varied signature sizes in order to attain differences in processing speeds based on the signature and the random number generations required. We tested the program against three CUDA-enabled machines with varying GPU capabilities, with one machine having two different GPU cards installed.

## Results

**Figure 1:**
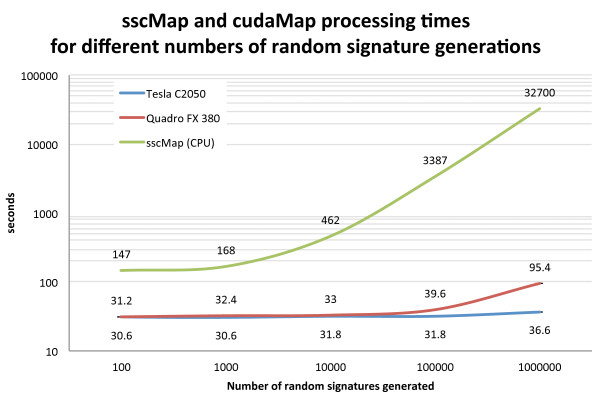
**sscMap and cudaMap processing times.** Performance of cudaMap using two separate GPUs versus sscMap, running on the same computer (Core i7) for increasing numbers of random signature generations. The signature length was 10 genes in all cases.

**Figure 2:**
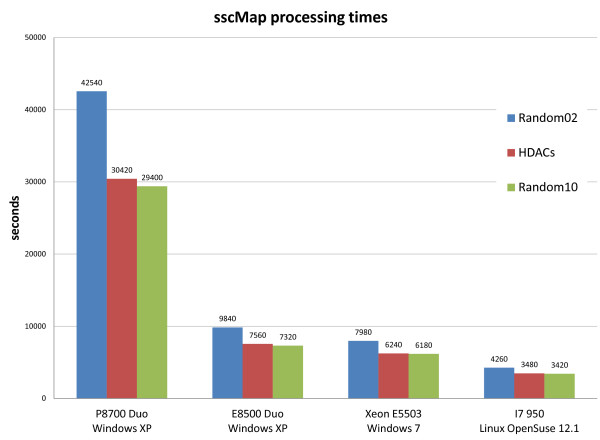
**sscMap processing times on different systems.** Performance of sscMap on different test systems for three signature queries of lengths 189 (Random02), 25 (HDACs) and 10 (Random10). 100 000 random signature generations were used in all cases.

**Figure 3:**
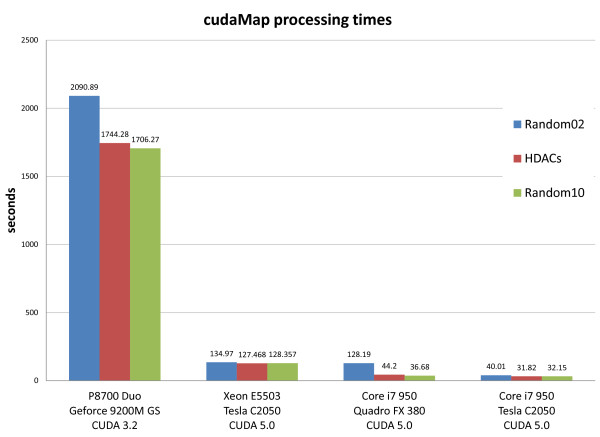
**cudaMap processing times on different systems.** Performance of cudaMap on different test systems for three signature queries of lengths 189 (Random02), 25 (HDACs) and 10 (Random10). 100 000 random signature generations were used in all cases.

To test the scalability of cudaMap, we ran it on several systems with different processor specifications, operating systems and GPUs, comparing the results to that of running sscMap. Figure 1 shows the outcome of running sscMap and cudaMap on an OpenSuse 12.1 Linux machine with an Intel i7 processor and 24GB of RAM and two GPU cards (Tesla C2050 and Quadro FX 380). The gene signature used consisted of 10 gene identifiers. The number of random gene signatures to generate for each p-value estimation was varied to compare the performances with increasing computational load. Figure 2 shows the processing times of running sscMap on the CPUs of the four machines listed in Table 1. Three gene signatures (with lengths n=10, 25, and 189 respectively) were tested with the number of random gene signatures per p-value set at 100 000. Figure 3 reports the processing times for the same set of tasks, but run with cudaMap on the three machines with GPU cards. As can be seen from these figures, with the 10-gene signature and 100 000 random signature generations per p-value estimation, the time taken to run sscMap on the Intel Core i7 processor was 57 minutes or 3387 seconds (the green curve in Figure 1). For cudaMap, even the least powerful test system, a laptop equipped with a Geforce 9200M GS GPU, was able to reduce the processing time of a similar task to 28 minutes or 1706 seconds (the first green bar in Figure 3), demonstrating the effectiveness of parallelization. Performance was greatly improved further by using faster GPUs, such as the Quadro FX 380 and Tesla C2050 cards tested, both of which reduced processing times to 30–40 seconds (Figure 1) for the same task. Furthermore, this improvement was maintained when up to 1 000 000 random signatures were generated per p-value (Figure 1). Results returned from cudaMap for all signatures had exact agreements with the sscMap results in terms of their connection scores. In terms of their p-values, agreements were achieved with regard to calls on statistical significance in all cases, except a few borderlines around the threshold p-value. Unlike the connection scores, exact agreement between p-values are not expected due to the way p-values were estimated, ie, by generation of random numbers.

The results here showed dramatic speedups in performance across all CUDA capable devices.

The quickest time was just over 30 seconds to perform an analysis utilising the Tesla C2050 card, which represented a near 20x speed up over the sscMap software running on the CPU of the same computer. The comparison of the two GPU cards on the same machine also shows the scalability of GPU computing, and in contrast to the i7 processor, suggests that using cudaMap to run a 10-gene signature with the standard 100 000 random signature generations did not tax either card too severely (the purple and blue curves in Figure 1). Increasing beyond this point (100 000 random signature generations), we start to see the difference between the two GPU cards, although the performance remained acceptable in both cases. sscMap, which runs with the CPU, on the other hand, showed dramatic increases in processing time to the point where it would become unrealistic to run the analysis with a large number of random signatures to generate. In general, higher accuracy of p-value estimation is achieved when more randomizations are used, which is feasible when running cudaMap but may not be with sscMap because of the computation time required.

## Conclusions

The cudaMap software is open source and free to use with parallel computation harnessing local GPUs. It carries out gene expression connectivity mapping tasks in very fast turnaround times with the functionality of running multiple signatures. Our tests demonstrated that cudaMap even running on a laptop with a small compute capability (Geforce 9200M GPU) could outperform sscMap running on a high-spec desktop computer such as the Linux machine with an i7 CPU. In most cases and for most signature sizes cudaMap performs under the 40 second mark on this machine using its NVIDIA Tesla card, a dramatic reduction in computing time compared to running sscMap on the same machine. With its capability of fast gene expression connectivity mapping for robust candidate therapeutics selection, cudaMap can serve as a useful tool and resource in modern cancer research, where high throughput ‘omics’ technologies are playing indispensable roles. As the ‘omics’ datasets continue to grow in their sizes and information content, each of these datasets will, in turn, require more sophisticated analysis and demand more computing power. The development of cudaMap represents an effort to meet the computational demands of a popular bioinformatic procedure in biomedical research, namely gene expression connectivity mapping to establish connections among genes, diseases and drugs. We believe this accelerated software will be a valuable tool and resource in many areas of biomedical research including drug discovery, drug re-positioning, and biological phenotypic targeting with small-molecule compounds.

## Availability and requirements

**Project name:** cudaMap**Project home page:**http://purl.oclc.org/NET/cudaMap**Operating system(s):** Windows and Linux**Programming language:** C/C++/CUDA**Other requirements:** CUDA 3.2 and above toolkit**License:** Creative Commons license by-nc 3.0.**Any restrictions to use by non-academics:** For commercial use, please contact the authors

## Competing interests

The authors declare that they have no competing interests.

## Authors’ contributions

DMA, PH and SDZ designed the study. DMA and PB wrote the software and tested the implementation and PD interpreted and analysed the data. All authors contributed to writing the manuscript and technical input. All authors read and approved the final manuscript.

## Supplementary Material

Additional file 1 Pseudocode for the key algorithms used in sscMap and cudaMap. Click here for file
