# Supplementary material for: cudaMap: a GPU accelerated program for gene expression connectivity mapping
Source: BMC Bioinformatics. 2013 Oct 11;14:305. doi: 10.1186/1471-2105-14-305 (PMC3852931; doi:10.1186/1471-2105-14-305)
Supplement: Additional file 1 — Pseudocode for the key algorithms used in sscMap and cudaMap. [file 1471-2105-14-305-S1.pdf]

Description of the algorithms:

N is the total number of genes (or interchangeably probes) on the microarray platform used.
An index from 0 to N-1 uniquely specifies one of the N genes.

R is a reference gene expression profile
It is represented by a 1D integer array of length N
The array index specifies the gene, while the integer value gives the signed rank of the gene

T is a refset, ie, a set of n individual reference profiles; n is also referred to as setSize

s is a query gene signature of length m

where m is the number of genes included in the gene signature

A gene signature is represented by two integer arrays of length m.
One array contains integers from 0 to N-1, indicating which genes are included in this gene signature
The other array contains +1 or -1, indicating the up- or down- regulation status of the corresponding genes.

N_rdmsigs is the total number of random signatures for each p value estimation, which is usually set as 10,000 or 100,000.

The following pseudo code describes the key process in sscMap

```
For (each refset T) {
      For (each query signature s){

            observed_score = connection score between T and s according to Eq(6) in Zhang&Gant2008

            // allocate a real-valued array to hold N_rdmsigs random scores
            float randomscores[N_rdmsigs]


            ////////////////////////////////////
            For(i in 1:N_rdmsigs){

                  generate a random gene signature si, represented by two integer arrays of length m,
                      One array contains random indexes from 0 to N-1, to indicate
                      which genes have been selected into this random gene signature.
                      The other array contain +1 or -1, randomly selected to indicate the up- or
                      down- status of the corresponding gene.

                  randomscores[i]= the connection score between T and si according to Eq(6)Zhang&Gant2008


            }
            ////////////////////////////////////

            compare observed_score against randomscores to get the p value

      }// end of For (each query signature s) loop
}// end of For (each refset T)
```

As can be seen from the pseudo code above, the generation of N_rdmsigs random gene signatures and the calculation of the connection score between each random signature and the refset are carried out sequentially in sscMap. As N_rdmsigs is a very large number (100K normally), this is the most time consuming part of the sscMap program.

In cudaMap, this part of the task (highlighted in red fonts above) is parallelised using a GPU device.

In cudaMap we create one random gene signature with m random real numbers U[0,1) (uniformly distributed between 0 and 1) using an algorithm essentially the same as the following.

```
X[m] is an array of random real numbers uniformly distributed U[0,1)
Status[m] is an array of integers +1 or -1, indicating the up- or down- regulation status of m genes
Indexes[m] is an array of integers specifying the indexes from 0 to N-1

For(k in 1:m){
      If(X[k]<0.5) Status[k]=1  // this gene is up-regulated
      Else Status[k]=-1          // otherwise it is down-regulated
      // X[k] is also used to select a gene from N
      X[k]=X[k]*2.0
      If(X[k]>=1.0) X[k]=X[k]-1.0
      Indexes[k]= integer (N*X[k])
}
```

To generate N_rdmsigs random gene signatures of length m, a total m*N_rdmsigs uniformly distributed real numbers are required.

The following pseudo code describes the algorithm for the key task

```
// allocate memory space on cuda device to hold a total m*N_rdmsigs random number U[0,1)
// allocate memory space on cuda device to hold N_rdmsigs random connection scores

float *device_randomValues, *device_randomScores;
CUDA_CHECK_RETURN(cudaMalloc((void **) &device_randomValues, sizeof(float) * m*N_rdmsigs));
CUDA_CHECK_RETURN(cudaMalloc((void **) &device_randomScores, sizeof(float) * N_rdmsigs));



// Create an array of uniformly distributed random numbers between 0 and 1,
// curandGenerator_t gen;
```

```
//  gen is a cuda random number generator, which had been defined and initialized

CUDA_CHECK_STATUS(curandGenerateUniform(gen, device_randomValues, m*N_rdmsigs));
// now device_randomValues holds an array of m*N_rdmsigs random U[0,1) real numbers.


//////////////////////////////////////////////////////////////////////
// Compute the connection scores of the N_rdmsigs random gene signatures with the Refset T
// The array device_refRanks stores the (summed) Ranks for the Refset T, which
// had been copied to the CUDA device

int blocksPerGrid = (int)ceil((float)N / (float)threadsPerBlock);
// const int threadsPerBlock = 128 had been defined elsewhere

computeRandomConnectionScores <<< blocksPerGrid, threadsPerBlock >>>(device_randomValues,
      device_refRanks , device_randomScores);

// see the pseudo codes below for more detailed steps within the function above
//////////////////////////////////////////////////////////////////////

CUDA_CHECK_RETURN(cudaThreadSynchronize());
// the N_rdmsigs random scores are now stored in the array device_randomScores


// copy these random scores from device memory to main memory
float randomScores[N_rdmsigs];
CUDA_CHECK_RETURN(cudaMemcpy(randomScores, device_randomScores,
      N_rdmsigs * sizeof(float), cudaMemcpyDeviceToHost));


// Then Compute the p-value
pValue = compare observed_score against the distribution of randomScores
```

The following pseudo code gives more details on the computing of random scores on the GPU

```
float temp = 0.0;
int idx = blockIdx.x * blockDim.x + threadIdx.x;
if (idx < N_rdmsigs) {
      int M=m* N_rdmsigs;
      for(int col = idx; col < M; col += N_rdmsigs) {
            float random = device_randomValues[col];
            float regulationStatus = 1.0;
            if (random >= 0.5) {
                  regulationStatus = -regulationStatus;
                  random = random - 0.5;
            }
            // now random is a real number in [0,0.5)
            // Scale up random to become an integer index < N
            int rangeInArray = __float2int_rd(random * N * 2);

            // Add the contribution to the connection strength
            temp += device_refRanks[rangeInArray] * regulationStatus;
      }

      // Normalizing by appropriate factors UCmax and setSize
      // UCmax is defined by Eq.(3) in Zhang&Gant2008; setSize refers to the n in Eq.(6).
      device_randomScores[idx] = temp / UCmax /setSize;

} //end of if (idx < N_rdmsigs)
```
